# Taking Electrons out of Bioelectronics: From Bioprotonic Transistors to Ion Channels

**DOI:** 10.1002/advs.201600527

**Published:** 2017-03-14

**Authors:** Xenofon Strakosas, John Selberg, Zahra Hemmatian, Marco Rolandi

**Affiliations:** ^1^ Department of Electrical Engineering University of California Santa Cruz Santa Cruz California 95064 USA

**Keywords:** bioprotonics, bioelectronics, biotic/abiotic, protons, palladium

## Abstract

From cell‐to‐cell communication to metabolic reactions, ions and protons (H^+^) play a central role in many biological processes. Examples of H^+^ in action include oxidative phosphorylation, acid sensitive ion channels, and pH dependent enzymatic reactions. To monitor and control biological reactions in biology and medicine, it is desirable to have electronic devices with ionic and protonic currents. Here, we summarize our latest efforts on bioprotonic devices that monitor and control a current of H^+^ in physiological conditions, and discuss future potential applications. Specifically, we describe the integration of these devices with enzymatic logic gates, bioluminescent reactions, and ion channels.

## 1 Introduction

Understanding biological processes ranging from enzymatic reactions to whole organ functions is of importance for biology and healthcare. In many of these biological processes ionic currents and fluctuations in ionic concentration play a central role. The field of bioelectronics couples electronic devices with biology to record and modulate these processes.^1^, ^2^, ^3^, ^4^, ^5^, ^6^ Differences in methods of communication (electrons versus ions) between traditional electronic devices and biological systems result in a challenge at the interface.^7^, ^8^ Silicon nanowire and carbon nanotube transistors integrated with enzymes, antibodies, and lipid bilayers use ions to gate electronic currents and record biological reactions in the intracellular and extracellular space.^2^, ^3^, ^9^, ^10^, ^11^, ^12^, ^13^, ^14^, ^15^, ^16^ Organic polymers with mixed electronic and ionic conductivity integrated in electrodes and electrochemical transistors transduce ionic to electronic currents and amplify small biological signals^3^, ^17^, ^18^, ^19^, ^20^, ^21^, ^22^ In addition, organic iontronics locally deliver ions and neurotransmitters in the extracellular space to affect cell and tissue function.^23^, ^24^, ^25^, ^26^ Batteries with biocompatible materials are used for giving energy to these systems.^27^ Other approaches, such as optogenetics use light triggered ion channels expressed in cell membranes to manipulate cell function.^28^, ^29^, ^30^, ^31^, ^32^, ^33^ These innovative devices open up a window to study biological processes and improve current biomedical tools and techniques.^13^

**Figure 1:**
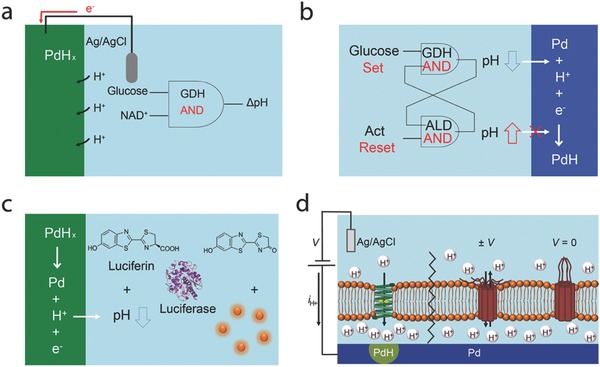
Bioprotonic devices interfacing biology by monitoring and controlling reactions. a) A Pd/PdH_x_ contact is controlled by enzyme logic pH modulation. Reproduced with permission.^51^ Copyright 2015, the authors. Published under CC‐BY 4.0 license. b) Schematic of the enzymatic flip flop circuit as a bi‐stable pH modulating system controlled by logically processed biochemical signals. c) Control of firefly bioluminescence reaction with pH modulating bioprotonic transducer. Reproduced with permission.^52^ Copyright 2016, the authors. Published under CC‐BY 4.0 license. d) integration of a protonic device with a lipid bilayer and ion channels. Reproduced with permission.^53^ Copyright 2016, the authors. Published under CC‐BY 4.0 license.

Along with ions and small molecules, protons (H^+^) play an important role in biology. H^+^ are involved in many biological processes, including oxidative phosphorylation,^34^ proton gated ion channels,^35^, ^36^ and pH regulation.^37^, ^38^ Devices that monitor and control H^+^ currents facilitate the interaction with these processes. Inspired by electronic and semiconducting devices, our group has developed analogous bioprotonic devices that conduct H^+^ and OH^–^ instead of electrons and holes. These devices include complementary bioprotonic transistors,^39^ diodes,^40^ and protonic resistive memories.^41^ At the heart of these devices are Palladium (Pd) and Palladium hydride (PdH_x_) contacts that are able to transduce a current of H^+^ and OH^−^ into an electronic current at the contact proton conducting interface.^39^ These devices are already described in topical reviews.^42^, ^43^ We also used Pd contacts to measure the protonic conductivity in biological materials such as melanin^44^ and the jelly found in the ampullae of Lorenzini of sharks and skates — the highest naturally occurring proton conductor.^45^ Gorodestky and co‐workers have demonstrate high proton conductivity in reflectin squid proteins and transistors.^46^, ^47^, ^48^, ^49^ Geim and co‐workers used PdH_x_ contacts to probe the H^+^ conductivity across graphene.^50^ In this review, we summarize our recent efforts on developing bioprotonic devices that control H^+^ currents at the PdH_x_ contact solution interface to monitor and control biological reactions (**Figure** **1**a–d).^51^, ^52^, ^53^, ^54^, ^55^

## 2 H^+^ Transfer at the Contact‐Liquid Interface

**Figure 2:**
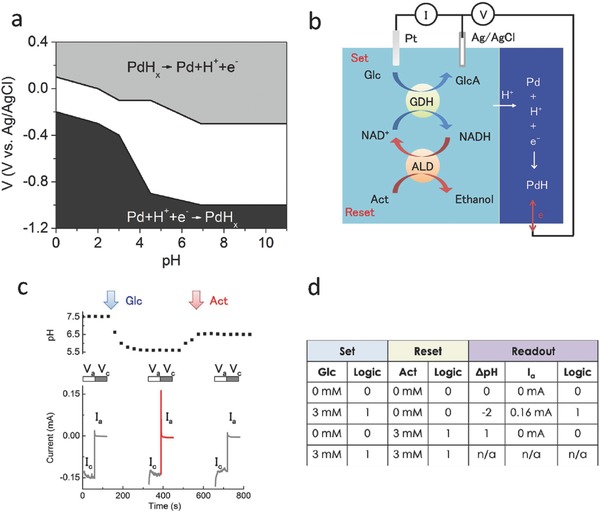
Bioprotonic platform monitors enzymatic reactions. a) Voltage dependence of H^+^ across the Pd/PdH_x_ solution interface as a function of pH. Reproduced with permission.^51^ Copyright 2015, the authors. Published under CC‐BY 4.0 license. b) Enzymatic flip‐flop with glucose dehydrogenase (GDH) and Alcohol dehydrogenase (ALD) integrated with the PdH_x_ biotransducer. The electrochemical setup has a Pd working electrode, Ag/AgCl reference electrode and Pt counter electrode. c) Upper panel shows the pH change when adding glucose and acetaldehyde into the solution. Addition of glucose drops the pH from 6 to 5.5 and addition of acetaldehyde brings the pH back to 6. Lower panel shows I_c_ (transfer of H^+^ to Pd) and I_a_ (transfer of H^+^ to solution) in response to pH change. V_c_ = 0 V and V_a_ = −0.95 V is applied during the process respectively. I_a_ is negligible when solution pH is above pH 6. pH 5.5 induced by the addition of Glucose into the solution causes I_a_ = 0.16 mA. The return of pH to above 6 with the addition of Act results in no I_a_. d) Truth table of the set‐reset enzymatic flip‐flop circuit. Reproduced with permission.^52^ Copyright 2016, the authors. Published under CC‐BY 4.0 license.

Miyake et al. studied H^+^ transfer at the Pd/PdH_x_ interface in aqueous environments.^51^ In common electrolytes, H^+^ exist as hydronium ions (H_3_O^+^). When a Pd contact is immersed in solution and is biased at a negative voltage versus a reference electrode, H^+^ (in the form of H_3_O^+^) adsorb onto its surface. Upon adsorption, a H^+^ is reduced to H with an electron from the Pd. The H subsequently diffuses into the Pd contact and forms PdH_x_; with *x* up to 0.6–0.7.^56^, ^57^ Pd expands during absorption of H to form PdH_x_ and the associated mechanical stress may result in degradation of PdH_x_ /proton conducting material contact.^58^ In our proton conducting synaptic transistors and memories,^41^ we were able to run the devices through several cycles of Pd/PdH_x_ transformation without obvious degradation. Longer durability studies are necessary to further verify long‐term performance. The overall equation for this reaction is Pd + H_3_O^+^ + e^–^ → PdH_x_ + H_2_O, in which a H^+^ transfers across the Pd/PdH_x_ solution interface. The electronic current at the Pd contact arising from the e^–^ transfer from the Pd to the H_3_O^+^ is monitored with an external circuit as I_H+_.^51^ The threshold voltage applied to the Pd contact for the net transfer of H^+^ across the Pd/PdH_x_ solution interface depends on the pH of the solution due to the influence of [H^+^] on the protochemical potential (**Figure** **2**a). As expected, in acidic solutions with high [H^+^] and protochemical potential, a small negative voltage applied on the Pd contact (V = –0.2 V) is adequate to transfer H^+^ from the solution to the Pd contact and form PdH_x_. In basic solutions with low [H^+^] and protochemical potential, a larger negative voltage (V = –0.9 V) applied on the Pd contact is required to transfer a H^+^ from the solution to the Pd contact and form PdH_x_.^58^

As a proof‐of‐concept, our group used the pH dependence of the behavior of these Pd/PdH_x_ devices to monitor enzymatic reactions.^51^, ^52^ Specifically, we monitored the conversions of glucose to gluconic acid, and acetaldehyde to ethanol, catalyzed by the enzymes glucose dehydrogenase (GDH) and alcohol dehydrogenase (ALD) respectively in the presence of a cofactor nicotine adenine dinucleotide (NAD^+^) (Figure 2b). The conversion of glucose to gluconic acid drops the pH to a level that favors the transfer of H^+^ from the solution to the Pd contact and consequent formation of PdH_x_. When a positive voltage is applied to the PdH_x_ contact, the subsequent transfer of H^+^ from the PdH_x_ back into the solution results in significant positive I_H+_ that we use as indication that the enzymatic reaction occurred (Figure 2c). The enzymatic reaction requires both glucose and NAD^+^ (two inputs) equivalent to an AND logic gate. ALD and acetaldehyde increase the pH feeding back into the enzyme logic system. High pH no longer favors the transfer of H^+^ from the solution to the Pd contact and changes the output of the system. This becomes effectively an enzymatic flip‐flop with the input and output values displayed in Figure 2d. A flip‐flop is a latch circuit with two stable states (set and reset) that can be used to store information. In the enzymatic flip‐flop, addition of glucose results in a “set” state, while addition of acetaldehyde results in a “reset” state. This is an example of a biosensor, in which Pd in contact with an electrolyte is able to monitor enzymatic reactions using pH as a marker.

**Figure 3:**
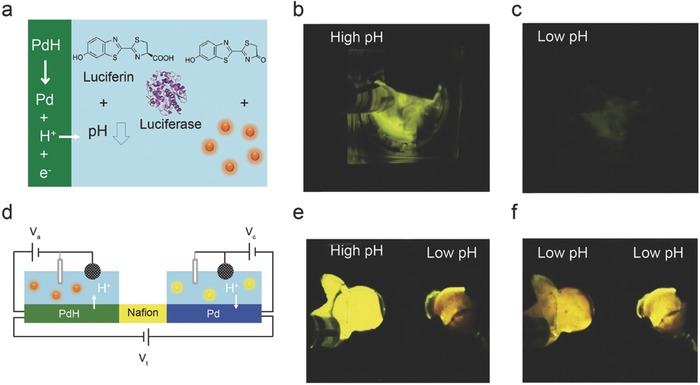
Bioprotonic devices controlling biological reactions: a) Schematic of firefly bioluminescence reaction integrated with pH modulating biotransducer. Luciferin is oxidized into oxy‐luciferin in the presence of the luciferase enzyme. The reaction emits light. The solution pH affects the color of the light emitted. When the PdH_x_ modulates the pH, the bioluminescence light in the solution changes. b–c) Bioluminescence turned on and off by the pH modulator. The initial pH of solution is low (pH = 5.8). The Pd platform increases the pH to reach an optimal pH for the enzymatic reaction. Bioluminescence turns on. When the device is off, the solution pH returns to initial pH and bioluminescence is off. d) Schematic of the pH modulator circuit integrated with bioluminescence enzymatic reaction. e–f) Bioluminescence changes color when the pH drops. Reproduced with permission.^52^ Copyright 2016, the authors. Published under CC‐BY 4.0 license.

A voltage applied to the Pd/PdH_x_ contact controls transfer of H^+^ between the solution and the Pd/PdH_x_ contact. As a result, we are able to change the [H^+^] and the pH of a solution with bioelectronic means. We studied the effect of bioelectronic pH modulation on the catalytic reaction of luciferase, an enzyme responsible for the yellow‐green glow of the firefly (**Figure** **3**a).^52^ Luciferase catalyzes the pH sensitive and ATP dependent conversion of luciferin to oxyluciferin, resulting in the emission of a photon.^59^ The reaction is favorable in alkaline conditions (pH = 8) and less so in acidic conditions (pH = 6). For bioelectronic control of the reaction, luciferase and luciferin were integrated with a Pd/PdH_x_ contact that transferred H^+^ from and to solution changing solution [H^+^] and consequently pH. When a negative voltage is applied to the Pd (V = –0.9 V for pH = 6.8) and inducing the transfer of H^+^ from the solution to the Pd contact, the pH of the solution increases. At higher pH, luciferase catalyzes luciferin into oxyluciferin at higher rates with bright bioluminescence as the output (Figure 3b). When the voltage applied to the Pd/PdH_x_ contact is switched to zero, H^+^ are released back into the solution thus lowering the pH. At lower pH, the enzymatic reaction of luciferase is quenched and the bioluminescence output decreases (Figure 3c). This cycle is reproducible until either the substrate luciferin or the ATP required for the enzymatic reaction are fully consumed. One consequence of this device configuration is that H^+^ need to be loaded from the solution to the Pd contact and form PdH_x_ first in order to be transferred back into the solution. In this fashion, the solution pH can be lowered to its initial value and not further.

To address this limitation, we developed a more advanced circuit with two Pd electrodes in two separate chambers connected with Nafion as a proton conducting membrane.^52^ In this device, one chamber is used as reservoir to load H^+^ into the corresponding Pd/PdH_x_ contact, which is then used to transfer the H^+^ to the Pd contact in the other chamber across the proton conducting membrane (Figure 3d and e). As a result, pH is decreased in the accepting chamber and the resulting bioluminescence output is weaker (Figure 3f). This platform acts as a H^+^ pump, similarly to the ion pump,^23^ and it has the ability to modulate the pH in both directions without replacing the electrolyte. The ability of bioelectronics to use the control of pH in solution to modulate the activity of enzymes and ion channels can provide a new biomedical tool for treatment of diseases. One potential application is interfacing acid‐sensing ion channels (ASICS) that exist in the central and peripheral nervous system.^36^, ^60^

## 3 Integration of Bioprotonics with Ion Channels

Ion channels mediate communication of cells with the external world and each other.^61^ To increase the functionality of the bioprotonic devices, we integrated ion channels with Pd/PdH_x_ and modulate H^+^ currents and pH gradients across phospholipid membranes.^53^ These devices incorporate a supported lipid bilayer (SLB) at the Pd/PdH_x_‐solution interface to mimic the cellular membrane. The SLB provides a self‐repairing support for the integration of ion channels and splits the solution into bulk and isolation layers. The SLB was functionalized with three different ion channels gramicidin, alamethacin,^53^ and deltarhodopsin.^54^

**Figure 4:**
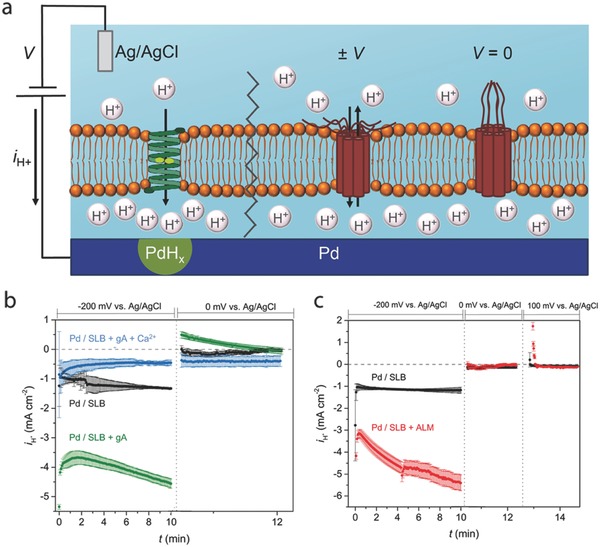
A bioprotonic device at the interface with ion‐channels. a) Schematic depiction of the bioprotonic device. (left) A bioprotonic device with integrated Gramicidin (gA) supports the flow of H^+^ across the SLB upon application of a negative voltage. When H^+^ reach the surface of the Pd contact, they are reduced to H by an incoming electron and diffuse into the palladium to form a hydride (PdHx). A reduction current is measured as –I. (right) a bioprotonic device with integrated Alamethicin (ALM). A negative or positive voltage above threshold value opens the gate and allows H^+^ flow across the SLB, turning the device ON. At V = 0 mV, no H^+^ flows across ALM and the device switches OFF. b) I‐t plot for gA at V = –200 mV and V = 0 mV. Black trace SLB, green trace SLB + gA, blue trace SLB + gA blocked by Ca^2+^. c) I‐t plot ALM at V = –200 mV and V = 0 mV and V = 100 mV. Black trace SLB, red trace SLB + ALM. Reproduced with permission.^53^ Copyright 2016, the authors. Published under CC‐BY 4.0 license.

Gramicidin (gA) is a constantly open (ON) ion channel found in bacteria that permeabilizes a membrane to small monovalent cations.^62^ A negative voltage (–0.2V) applied to the Pd contact that is functionalized with SLB and gA results in a much higher I_H+_ than when the contact is functionalized with the SLB alone (**Figure** **4**a–b). This I_H+_ corresponds to H^+^ transfer from the solution to the Pd contact and subsequent formation of PdH_x_. Transfer of H^+^ from PdH_x_ across the gA‐SLB back into the bulk solution occurs when the voltage is set to 0V on the PdH_x_ contact. The resulting I_H+_ indicates that the initial transfer into the Pd occurs. gA channels are switched OFF chemically with the addition of Ca^2+^ ions that block the gA channel and inhibit H^+^ transfer across the SLB from the solution into the Pd contact (Figure 4b).

Alamethicin (ALM) is a voltage‐gated ion channel that is open above a threshold transmembrane voltage of 0.1V.^63^ We demonstrated bidirectional voltage‐gated switching of I_H+_ across an SLB functionalized with ALM (Figure 4c). A negative voltage (–0.2V) applied to the Pd contact drives I_H+_ through the open ALM into the Pd contact to form PdH_x_. In contrast to gA, no I_H+_ from the PdH_x_ to the bulk solution occurs when the voltage is set to 0V, this corresponds to a closed state (OFF) of ALM. A positive voltage of 0.1V sets the channel to an open state (ON) and an I_H+_ into the bulk occurs. The gating of I_H+_ is a signature of the voltage dependence of ALM and is consistent with prior work.

**Figure 5:**
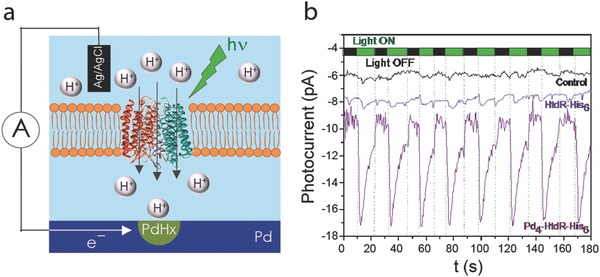
Light‐activated conversion of protonic to electronic current a) Schematic showing the operating principle of the device. b) The photocurrent response of devices prepared with DOPC control liposomes (black trace), HtdR‐His6 (blue) or Pd4‐HtdR His6 proteoliposomes (purple) was recorded while the Pd contact was kept at an applied voltage of −50 mV versus Ag/AgCl and exposed to consecutive 10 s illumination cycles with a 523 nm LED. Reproduced with permission.^54^

We demonstrated optical control of H^+^ currents with deltarhodopsin (HtdR) integrated in the SLB and the Pd contacts.^54^ HtdR is a light triggered archaeal proton pump (**Figure** **5**a). Upon illumination of the HtdR‐SLB, we observed a rapid current response corresponding to the HtdR pumping action of a H^+^ across the SLB into the Pd contact and subsequent PdH_x_ formation. This spike in the H^+^ photocurrent was repeatable over multiple illumination cycles with 523nm light (Figure 5b).

Combining electronic devices and ion channels to enhance our interaction with biology provides many options for research in the future. The creation of multi‐ion‐channel systems capable of more complex logic functions and increased functionality are possible. A further‐off goal is to utilize similar devices to someday exhibit control over processes within a cell.

## 4 Conclusion

In bioelectronics, several strategies exist for devices and materials to couple with biology (summarized in **Table** **1**). We have developed bioprotonic devices that can monitor, record, and modulate H^+^ currents that are product of or affect biological reactions. At the heart of these devices is the use of the Pd/PdH_x_ couple as a transducer of H^+^ currents into electronic currents and vice versa. The use of Pd/PdH_x_ in bioelectronics has open the doors to new types of applications and devices including enzyme logic, control of bioluminescence, and platforms for integration of ion channels toward intracellular communication. H^+^ currents and pH gradients affect biological reactions and cell function.^61^ For example, In the central and peripheral nervous system, excessive neuronal activity decreases pH due to metabolic production of CO_2_, in turn low pH decreases neuronal excitability.^36^ Other examples include enzymatic function,^52^ acid sensitive ion channels,^60^ and proton pumps.^54^ In a broad spectrum of applications, bioprotonic platforms that monitor and control pH can interface cell functions, enzymatic reactions, and can potentially be used as biomedical devices for diagnostics and treatment.

**Table 1:** Table summarizing communication carriers, materials, and target applications for bioelectronic devices

Field	Charge carriers	Materials	Applications
Bioelectronics	Electrons	Metals (Au,Pt,Ti,)	Ionic to electronic signal transduction, electrical stimulation.
Organic bioelectronics, Iontronics	Electrons, Ions, small molecules	Conducting Polymers (PEDOT:PSS), Ionic membranes (NaPSS, Chitosan) Specific to ions that exist in the reservoir electrolyte	Ionic to electronic signal transduction (diagnostics). Delivery of ions and neurotransmitters (treatment).
Bioprotonics	Protons	Metals and polymers specific to H+ (Palladium, Nafion)	Monitor and control of pH in enzymatic reactions, cell's function, ATP synthesis.
